# Remedies and Their Uses

**Published:** 1906-12-22

**Authors:** 


### Remedies and their Uses.

### Creosotal.

This substance is a carbonate of creosote, and is said to have an antipyretic as well as an antiseptic action. It may be prescribed (Kirsch) in a mixture thus:—

| | | |
|---|---|---|
| ℞ Creosotol | ... ... ... | 35 gr. |
| Sp. Vini Gall. | ... ... ... | ℥j. |
| Syr. | ... ... ... ad. | ℥ss. |

This may be given four times in 24 hours, either alone or shaken up with milk. Small doses may also be given to children, infants taking 4-grain doses every six hours.

This drug is said to be more useful in bronchitis and pneumonia than in tuberculosis. In pneumonia it does not appear to shorten the attack, but influences the severity owing to its bactericidal action. Most authors advise its being given early, and in large doses. It is prepared by Bayen of Elberfeld.

### Cresamine.

Just as there are three isomeric dioxybenzenes so there are three isomeric bodies in which one hydrogen atom in the benzene ring is replaced by hydroxyl (OH), and another by methyl ($CH_3$). These are the three cresols, and they have the formula $C_6H_4.CH_3.OH$. Cresamine is a combination of these three creosols with ethylene diamine $CH_2NH_2.CH_2NH_2$, containing 25 per cent. of each dissolved in water. The ethylene diamine is said to increase the antiseptic effect, and to act as a solvent of mucus, pus, and albuminous material. A 2 to 20 per cent. solution of cresamine may be used as a spray for 10 to 15 minutes daily in affections of the respiratory tract. It has been employed in this manner by Günzel and Barney in cases of tuberculosis of the lungs, and has seemed to do good. The substance is prepared by Merck, of Darmstadt.

### Trigemin.

This excellent drug represents a valuable combination of the effects of antipyrin and butylchloral hydrate, being actually dimethyl-amido antipyrin butylchloral hydrate. It is particularly effectual in facial neuralgias, especially those of the fifth nerve; but its usefulness is by no means limited to this one variety of neuralgia. Many forms of headache, especially the occipital type, yield to its administration, and it has been found to give relief in those most painful eye-affections—glaucoma and irido-cyclitis. Latterly, too, it has been given with marked relief in primary dysmenorrhœa. It will fail, however, in cases of active inflammation, producing no effect in acute rheumatism, nor in any disease attended by pyrexia, when it is badly tolerated. The preparation should always be of a pure white colour, since it rapidly decomposes and turns brown when it has been exposed to the air. It possesses a very unpleasant taste and occasionally irritates the gastric mucous membrane. The best form for administration is in capsules containing 3 or 4 grains, which may be repeated after five or six hours. Apparently the drug does not lose its effect upon individual patients by prolonged use.

